# Emerging Putative Biomarkers: The Role of Alpha 2 and 6 Integrins in Susceptibility, Treatment, and Prognosis

**DOI:** 10.1155/2012/298732

**Published:** 2012-07-31

**Authors:** James R. Marthick, Joanne L. Dickinson

**Affiliations:** Menzies Research Institute Tasmania, University of Tasmania, 17 Liverpool Street Hobart, TAS 7000, Australia

## Abstract

The genetic architecture underpinning prostate cancer is complex, polygenic and despite recent significant advances many questions remain. Advances in genetic technologies have greatly improved our ability to identify genetic variants associated with complex disease including prostate cancer. Genome-wide association studies (GWASs) and microarray gene expression studies have identified genetic associations with prostate cancer susceptibility and tumour development. The integrins feature prominently in both studies examining the underlying genetic susceptibility and mechanisms driving prostate tumour development. Integrins are cell adhesion molecules involved in extracellular and intracellular signalling and are imperative for tumour development, migration, and angiogenesis. Although several integrins have been implicated in tumour development, the roles of integrin *α*_2_ and integrin *α*_6_ are the focus of this paper as evidence is now emerging that these integrins are implicit in prostate cancer susceptibility, cancer stem cell biology, angiogenesis, cell migration, and metastases to bone and represent potential biomarkers and therapeutic targets. There currently exists an urgent need to develop tools that differentiate indolent from aggressive prostate cancers and predict how patients will respond to treatment. This paper outlines the evidence supporting the use of *α*_2_ and *α*_6_ integrins in clinical applications for tailored patient treatment.

## 1. Introduction

The diagnosis of aggressive prostate cancer at an early stage is crucial for successful management; however, clinicians still lack the diagnostic tools to identify indolent tumours from those likely to be aggressive and with a propensity to metastasise. Currently, the prostate-specific antigen (PSA) test still remains a widely used marker of choice for diagnosis and monitoring the progression of prostate cancer [1]. Thus molecular markers that can characterise individuals with a genetic susceptibility to prostate cancer identify malignant potential, provide real-time tumour surveillance, and potentially offer therapeutic intervention represent a major focus of current research. The advent of high-throughput genetic mapping technologies has not only highlighted the heterogeneity and complexity of the disease but also identified key molecules driving prostate cancer development and progression, and these molecules include the integrins. 

The integrins represent a large family of cell surface receptors that are responsible for cellular adhesion and complex formation with ligands within the extracellular matrix (ECM). They are noncovalent, heterodimeric, trans-membrane compounds, which extend from within the cytoplasm, spanning the cellular membrane, into the ECM [2–4]. The structure of these integrins is highly conserved between higher and lower organisms and is thought to highlight their importance to multicellular organisms [5]. To date, 18 alpha and 8 beta subunits have been identified, which form 24 heterodimers. These subunits can, in some cases, be further subdivided into “variants” created by alternate mRNA splicing events [6]. 

Individual integrins can form multiple heterodimers; for instance, *β*_1_ can form 12 separate complexes. Most, however, form only one or two [2]. The collagen-binding integrin *α*_2_ binds only with *β*_1_ and the laminin-binding *α*_6_ only with *β*_1_ and *β*_4_. Many integrins can be considered promiscuous as they can have multiple ligands; for example, tenascin, collagen, and laminin are all ligands for *α*_2_. Integrin promiscuity perhaps reflects the need to initiate different cellular processes using the same available ECM proteins [2]. For example, the ligand fibronectin binds to both *α*_5_ *β*_6_ and *α*_5_ *β*_1_, where *α*_5_ *β*_6_ stimulates cell migration and *α*_5_ *β*_1_ suppresses it [7, 8]. The ability of integrins to bind to multiple ligands is thought to be an advantage when the response is more important than the ECM protein signalling it, for example, in wound healing [9]. 

Integrins are key mediators in a number of cellular processes including cell survival, proliferation, cell migration, angiogenesis, and lymphangiogenesis [10]. Cell migration and motility are crucial to maintain and promote healthy cell development, wound healing and immunity. They are complex processes requiring tightly regulated and coordinated intracellular signal transduction with the ECM. The aberrant expression of key molecules, such as the integrins required for cell-to-cell and cell-to-matrix interactions, is hypothesised to lead pathogenic phenotypes, such as neoplasia and progression towards the metastatic phenotype [11]. Thus as modulators of cell adhesion, the integrins are now thought to play a key role in prostate cancer tumourigenesis. Furthermore, several of these integrins represent excellent therapeutic targets. Inhibitors targeting selected integrins have already reached Phase II and Phase III human trials for glioblastoma, lung, and breast cancers [12]. In addition, work on therapeutics targeting the *α*_2_, *α*_6_, and *β*_4_ subunits has commenced, with several of these currently undergoing validation in human trials. 

The majority of deaths associated with prostate cancer are the direct result of metastatic disease, which is thought to arise as tumour cells escape via the basement membrane, through the prostate capsule, where they are transported throughout the body. These cells are then able to disseminate and propagate secondary lesions [13]. Integrins provide the traction for tumour cell invasion by interacting with the extracellular matrix [14]. The invasion process arises via the breakage of actin filaments at the leading edge of the cell migratory path, which leads to dissociation between the cell and the extracellular matrix proteins [15], pointing to a critical role for integrins in this process. Over 80% of prostate cancer deaths incur metastases to bone [16]. The *α*_2_ and *α*_6_ integrins are the primary ligands for collagen and laminin, which are found abundantly in bone, and these integrins therefore are of interest in prostate tumour progression. 

The development of the pathogenic phenotype is thought to arise via *α*_2_ integrin eliciting the decay of ECM proteins, which is regulated by matrix metalloproteinases (MMPs). Ivaska and Heino [2] outline an interesting hypothesis by which MMP-1 and -2 mediated by integrins are able to degrade collagen. According to their hypothesis *α*_2_ *β*_1_ integrin binding to the fibrillar collagen matrix elicits the production of MMP-1, which degrades the collagen to gelatin, exposing an aspartate-glycine-arginine (RGD) site. The RGD site is then recognised and bound by *α*_v_ *β*_3_ which results in the upregulation of MMP-2 further degrading the gelatin matrix thus completing the process [2]. Supporting this theory is that cells expressing collagen-binding integrins *α*_2_ *β*_1_ and *α*_2_ *β*_2_ display marked changes in expression in response to collagen [17], thus highlighting the intrinsic relationship between these integrins and ECM ligands.

## 2. Gene Expression in Tumours

Expression of *α*_2_ and *α*_6_  integrin genes appears to be integral in prostate cancer progression. Studies of hormone refractory prostate cancer clearly demonstrate that *α*_2_ *β*_1_ has a major role in prostate cancer cells adhering to the bone matrix [18, 19]. The ability of *α*_2_ *β*_1_ to signal and interact with collagen in the bone microenvironment [20] may be particularly pertinent to the progression of prostate cancer. In normal prostate tissue, expression of *α*_2_ *β*_1_ is restricted to basal epithelial cells. During the normal differentiation of basal cells to intermediate cells, there is loss of substratum adhesion with an associated decrease in expression of integrins including *α*_2_ *β*_1 _[21]. 

Prostate basal epithelial cells also express the *α*_6_ subunit. Studies of *α*_6_ expression have reported that during progression from prostatic intraepithelial neoplasia to prostate carcinoma, *α*_6_ expression is maintained [22, 23], although *α*_6_ *β*_4_ expression diminishes and there is an increase in *α*_6_ *β*_1_. This increased expression is possibly due to a loss of *β*_4_ expression associated with differentiation. It is also known that, in prostate tumour development, the *α*_6_ subunit can be cleaved to a truncated form, *α*_6p_, which is no longer able to interact with the substratum [22]. Examinations of *α*_2_ *β*_1_ expression at different stages of prostate tumour development have concluded that *α*_2_ is initially downregulated in precursor lesions, leading to increased metastatic behaviour; however, variable aberrant expression patterns are observed [18, 21, 24–26].

Compelling evidence for *α*_2_ *β*_1_ as a modulator of tumour initiation and progression in prostate and breast cancer has recently been provided by Ramirez and colleagues [12]. A transgenic mouse model was employed to examine the role of *α*_2_ *β*_1_ in breast cancer, generating an *α*_2_ *β*_1_-null and MMTV-c-erbB2/Neu (mouse mammary carcinoma model) oncogene transgenic mouse cross. A significantly increased number of tumours and metastatic lesions were found in *α*_2_ *β*_1_-null cross mice, with an observed increased rate of tumour intravasation and increased numbers of circulating tumour cells compared with *α*_2_ *β*_1_-wild-type crosses. Ramirez and colleagues [12] also accessed public microarray data to validate their findings in human studies. Significantly reduced expression of *α*_2_ in breast carcinoma compared with normal breast tissue was demonstrated. Furthermore, it was revealed that reduced *α*_2_ expression correlated highly with the presence of metastatic lesions, poor prognosis, and overall reduced survival in breast cancer sufferers. Examination of the *β*_1_, *α*_3_, and *α*_1_ integrins showed no such associations [12]. A similar trend was observed in prostate tumour development with a progressive downregulation of *α*_2_ expressions associated with prostate tumour progression from the precursor lesion prostatic intraepithelial neoplasia (PIN), to carcinoma of the prostate and then to metastatic tumours.

A notable and repeated finding is that *α*_2_ is highly expressed by a subpopulation of cells in prostate tumours. Studies aiming to isolate subpopulations of tumour cells with “stem cell-like” properties have found that these “side-populations” express high levels of *α*_2_ *β*_1_ integrin [27]. In addition,**  **selected subpopulations of the PC-3 prostate cancer cell line, with stem cell-like activity and the ability to initiate serially transplantable tumours are also characterised by expression of high levels of the *α*_2_ *β*_1_  integrin [28]. Evidence suggests that these “cancer stem cells” (CSCs) are a subpopulation of CD44^+^ cells found within solid tumours [29]. Guzmán-Ramírez and colleagues [30] have also isolated cells from resected human prostate tumour tissue. Serial passaging of these tumour-derived cell populations was termed “prostaspheres” and resulted in a subpopulation with “stem cell like properties such as self-renewal and high clonogenic potential”; these cells were found to express high levels of both the *α*_2_ and *α*_6_ integrins. Additionally, in murine models, CSCs been shown to mediate EMT [29]. Prostate tumour stem cells displaying high levels of *α*_2_ *β*_1_ expression have also been described by Collins et al. [31] and Miki et al. [32]. These results suggest that there exists a subpopulation of tumour cells with dysregulated *α*_2_ and *α*_6_ integrin expression that perpetuate progression and tumour metastasis.

## 3. Integrins ***α***_2_, ***α***_6_ and the Genetics of Prostate Cancer

Age, race, and family history of prostate cancer remain the strongest risk factors for developing this disease. Recent advances in whole-genome analysis technologies have permitted the advent of high-density single nucleotide polymorphism (SNP) arrays for mapping and the use of genome-wide association studies (GWASs) to examine the underlying genetic contributors to prostate cancer. Since 2008, more than 15 GWASs have targeted prostate cancer, identifying more common susceptibility variants than that for any other cancer. A summary of the 46 GWAS variants thus far identified and their location is outlined by Goh et al. [33]. Whilst GWAS have to date identified more than 40 disease susceptibility variants, these 40 variants only explain approximately 30% of an individual's heritable risk of developing prostate cancer [34]. Thus a significant proportion of the genetic contributors to prostate cancer remains to be discovered. The phenomenon of “missing heritability” is not only relevant to prostate cancer, but is the current focus of genetic research in many complex diseases as well. Furthermore, GWAS are identifying common variants and these are generally of small to moderate effect size. This is true for most GWAS of complex disease; of the hundreds of variants discovered by GWAS, more than 80% lie outside of coding regions, and thus their functionality remains to be determined [35].

Attempts have been made to utilise known risk variants to develop polygenic risk calculators for prostate cancer with limited success, even when presence of family history is included in the model [36]. Zheng and colleagues [37] combined 16 known risk SNPs in five chromosomal regions and identified that, when combined with a family history, they were estimated to account for 46% of prostate cancer burden in a large Swedish cohort. More recently, Aly and colleagues [36] developed a logistic regression model based upon 35 GWAS identified variants, to determine whether a prostate biopsy should be collected for diagnosis. The model was able to reduce the number of biopsies required by 22.7% but was not able to distinguish between aggressive and nonaggressive prostate cancer [36]. In addition, Pashayan and coauthors [38] utilised 31 GWAS identified SNPs in a polygenic model and was able to reduce the number of men screened by 16%, but with a concomitant loss of sensitivity (3% increase in missed diagnoses). However, the model was able to identify younger men at increased risk. Our inability to identify the genetic risk factors explaining the majority of heritable risk is thus impeding our ability to develop accurate diagnostic tools to assist clinicians in the identification of clinically significant disease.

It is thought that “missing heritability” may be explained, at least in part, by rare variants. Rare mutations in the BRCA2 gene are already known to contribute to a small but significant proportion of prostate cancers with early-onset disease and poor survival [39, 40]. There has been a recent call to apply new genetic analysis technologies such as next-generation sequencing to familial studies as a powerful alternative approach for discovering the rarer variants likely to be of greater effect size associated with complex disease risk [41]. This approach has recently proven successful for several complex diseases including multiple sclerosis [42] and prostate cancer. Next-generation sequencing of a previously identified region of linkage on chromosome 17q21-22 has identified rare mutations in the HOXB13 gene associated with disease in familial prostate cancer [43].

Epigenetic alterations may also contribute to “missing heritability” associated with disease. Epigenetic phenomena such as heritable changes in DNA methylation, histone modifications, and chromatin remodelling can alter gene expression. Methylation at cytosine residues in CpG islands is well documented as influencing gene expression in numerous cancers [44], including prostate cancer [45, 46]. DNA methylation can aberrantly affect gene transcription by either preventing the transcriptional machinery from binding to the target region or by facilitating an interaction with chromatin remodelling proteins [47]. Unsurprisingly, the nature of these interactions is highly complex. Both global hypomethylation [48, 49] and hypermethylation of gene promoters are known to occur in prostate tumour development [50]. The methylation status of gene promoters has already been identified as predicative biomarkers with important clinical applications, for example, GSTP1 [51]. Whilst improved understanding of the role of heritable epigenetic modifications in prostate tumours is needed, the reversibility of epigenetic modifications and their propensity to arise before somatic mutations make them attractive targets for biomarkers and therapeutic intervention [47]. 

Most recently, emphasis has been placed on our inability to distinguish those aggressive tumours with a propensity to metastasise from more indolent disease that results in the unnecessary treatment of many men whose disease may never progress to clinical relevance. Given that current prostate cancer treatments such as prostatectomy, chemical castration, and radiation therapy are associated with considerable morbidities including impotence, incontinence, infection, and death, we urgently require a better understanding of the underlying drivers of this disease. The application of molecular screening techniques that can elucidate this subset of aggressive disease is therefore a focus of current translational research. Attempts to characterise SNPs associated with aggressive disease have produced conflicting results. Variants at 8q24 have been reported to be associated with increased risk of developing aggressive disease in a cohort of 823 Caucasian French men [52]. Furthermore, Helfand and colleagues [53] have also shown that the microsatellite loci (DG8S737) on 8q24 are significantly associated with aggressive disease (*P* = 0.04). Carriers of DG8S737 were significantly more likely to have a Gleason score greater than 7 and lymph node metastases [53]. FitzGerald and colleagues [54] also report that a variant on 15q13 (rs6497287) is significantly associated with aggressive prostate cancer (*P* = 0.004). While these studies represent important advances in our ability to screen for aggressive prostate cancer, they are currently unreplicated; therefore, the ability of these risk SNPs to accurately predict disease aggressiveness remains to be determined [33]. Issues also remain as to whether GWAS-identified genes are true cancer susceptibility genes or rather, those associated with tumour progression, as tumour detection requires a minimum size before it can be discovered [55]. We are yet to identify many of the true causal variants underlying the identified GWAS-identified SNP associations. Our lack of understanding of how these genetic associations drive prostate tumour development currently hampers translation of many of these genetic findings into the clinic. 

Genetic studies have previously identified polymorphisms residing within genes coding for the *α*_2_ and *α*_6_ subunits as significantly associated with disease, and gene expression profiling studies of prostate tumours have established altered gene expression in prostate tumours. For example, the GWAS conducted by PRACTICAL utilising over 30,000 cases and controls identified the variant rs12621278 in ITGA6 as significantly associated with prostate cancer susceptibility (allelic odds ratio = 0.75, confidence interval = 95%  *P* = 8.7 × 10^−23^) [56]. Interestingly, this variant has also been highlighted as playing an important role in prostate cancer progression. Cheng and colleagues [57] screened 26 SNPs previously identified by GWAS, in 788 patients that had undergone radical prostatectomies to test for an association with aggressive prostate cancer. Of these 26 SNPs, five were associated with aggressive prostate cancer progression, the strongest of which was rs12621278. The risk allele of this SNP increased the risk of prostate cancer progression by 2.4-fold (*P* = 0.0003) [57]. Genetic variants associated with prostate cancer have also been found in the ITGA2 gene which codes for the *α*_2_ integrin subunit. Utilising a familial linkage approach, FitzGerald and coauthors [58] identified a region on 5p13q12 in a large pedigree, with multiple cases of prostate cancer. Subsequently, two SNPs were identified, one in the 3′-UTR (rs3212649) and another synonymous mutation in exon seven (C807T), which increased prostate cancer risk both in the familial (OR = 2.16, CI = 1.19–3.92) and combined datasets (OR = 1.52, CI = 1.01–2.28). In addition, the C807T mutation has also been associated with an increased risk of developing oral and advanced breast cancers [59, 60]. There is also mounting evidence that silent mutations in coding regions are not as silent as once thought and are able to alter the rate at which protein translation occurs [61] or interfere with splicing events and RNA structure [62]. Whilst the C807T mutation does not change the amino acid sequence, it has been associated with changes in expression of the *α*_2_ receptor on the cell surface [63]. 

The integrins also feature prominently in microarray studies of prostate cancer tissue. Combining the results with GWAS-identified variants with observed changes in gene expression can provide insight into the functional significance of these variants. Gorlov and colleagues [55] performed a meta-analysis on gene expression data derived from normal and tumour tissue and combined this with GWAS data to identify overrepresented gene families. Genes coding for the *α*_2_, *α*_6_, and *β*_4_ integrins featured conspicuously amongst the most differentially expressed and most significantly associated with prostate cancer [55]. While the function of these variants remains to be determined, there is a large body of evidence supporting a role for these integrins in prostate tumour biology and therefore these integrins represent potential target candidate genes for use in diagnosis and prognosis. It is by gaining a thorough understanding of the genetic drivers of disease and their contribution to tumour biology that will permit effective translation of genetic findings in the clinical setting. 

## 4. Integrin ***α***_2_ ***β***_1_ as a Biomarker

The accumulated experimental evidence outlined above suggests that monitoring integrins, in particular *α*_2_ *β*_1_, may be useful in tracking prostate cancer development. The dual nature of cell surface receptor and differential expression in prostate cancer has contributed to the development of new imaging tools for tracking and identifying prostate cancer cells *in vivo*. Molecular imaging provides real-time data in a noninvasive and sensitive manner [64]. Optical imaging probes targeting the Asp-Gly-Glu-Ala (DGEA) motif in integrin *α*_2_ *β*_1_ with near-infrared-fluorescent (NIRF) imaging have been developed by Huang and colleagues [65]. A Cy5.5-conjugated DGEA peptide demonstrated high specificity in athymic mouse models with human PC-3 xenografts [65]. Crucially, the DGEA peptide worked exceptionally well as a ligand for *α*_2_ *β*_1_. However, the lack of quantitative data and challenges associated with optical imaging of deep tissues such as the prostate directed Huang and colleagues [65] to examine *α*_2_ *β*_1_ with nuclear imaging techniques. 

Positron emission tomography (PET) scanning is currently the most common method for cancer diagnosis; however, problems arise in prostate cancer imaging using traditional methods such as (18)F-fluorodeoxyglucose (FDG) potentially due to the often slow growing nature of the disease [66]. Utilising a (64)Cu-labelled *α*_2_ *β*_1_ probe and the previously developed DGEA peptide conjugated with a bi-functional chelator, the integrin *α*_2_ *β*_1_ probes were used in a PC-3 mouse xenograft model with some success. Tumours showed significant probe uptake with high specificity [66] and have since been validated in PC-3, CWR-22, and LNCaP prostate cancer cell lines [67]. Integrin-targeted nuclear imaging focusing on *α*_2_ *β*_1_ represents a significant advance in molecular imaging of prostate cancer. These advances potentially provide an excellent method by which prostate cancer progression may be better assessed in the clinical setting. Furthermore, imaging techniques such as this may be able to distinguish between different tumour subtypes in a heterogeneous disease, thus facilitating an ability to provide tailored therapies utilising anti-*α*_2_ *β*_1_ integrin therapies.

## 5. Integrin ***α***_2_ ***β***_1_, Diet, and Prostate Cancer 

Numerous epidemiological studies have sought to untangle the dietary components associated with risk of developing prostate cancer, and several of these have implicated lycopene as a potential protective agent these (reviewed by Giovannucci) [68], although some uncertainty remains [69, 70]. Lycopene is a powerful antioxidant, derived primarily from tomato-based products [71]. Intriguingly, malignant prostate cancer cell lines 22Rv-1, PC-3, and LNCaP display significantly reduced levels of *α*_2_ *β*_1_ when treated with lycopene [72]. In the same study, Buryeko and colleagues [72] also demonstrated that fish oil reduced *α*_2_ *β*_1_ expression in the more invasive cell lines LNCaP and PC-3. While the addition of lycopene and fish oil to cultured cell lines was able to reduce the expression of *α*_2_ *β*_1_, it was not able to attenuate the growth of the prostate cancer cell lines, suggesting that lycopene has an inhibitory role on migration and prostate cancer progression [72]. However, the use of lycopene and fish oils and chemopreventative agents remains controversial. Recently, clinical trials examining the efficacy of lycopene and fish oil in 84 men with low-risk prostate cancer use cDNA arrays after three months exposure [73]. However, no significant gene expression changes associated with taking lycopene or fish oil were identified [73].

## 6. Therapeutic Targets and Angiogenesis

The development of new blood vessels from preexisting ones requires interaction with the ECM [74]. Therefore, integrins can be viewed as a primary target for the prevention of angiogenesis associated with tumour progression. The role of integrins in tumour angiogenesis has been well described, with *α*_v_ *β*_3_, *α*_5_ *β*_5_, *α*_5_ *β*_1_, *α*_4_ *β*_1_, *α*_6_ *β*_1_, *α*_9_ *β*_1_, *α*_6_ *β*_4_, *α*_6_ *β*_1_, *α*_2_ *β*_1_, and *α*_1_ *β*_1_ all implicated in its pathology [14, 75]. The *α*_v_ and *α*_5_ integrins have been the focus of much of this research; however, the *α*_2_ integrins have also shown great promise as a target for antiangiogenic agents. 

Evidence suggests that angiogenesis is mediated by the vascular endothelial growth factor receptor pathway 1 (VEGFR1) and integrin *α*_2_ *β*_1_ [75]. Furthermore, *α*_2_ *β*_1_ is thought to be proangiogenic, with abrogation of the *α*_2_ *β*_1_ gene in murine models leading to decreased tumour size and vascularisation [75–77]. Unsurprisingly, the role of *α*_2_ *β*_1_ integrin in mediating angiogenesis is dynamic and involves crosstalk between different integrins and the cellular microenvironment. A number of inhibitory antibodies, bioactive molecules, and small protein fragments have been developed that aim to prevent angiogenesis by eliminating *α*_2_ *β*_1_ expression. Early attempts to abrogate angiogenesis via the VEGF pathway and *α*_2_ integrin utilised anti-*α*_2_ monoclonal antibodies. Nude mice with human squamous cell carcinoma xenografts displayed a 40% decrease in tumour growth and a reduction in angiogenesis by 60% when treated with an anti-*α*_2_ antibody [78]. Furthermore, the anti-*α*_2_ antibody HA1/29 reduced endothelial cell migration in an immobilized collagen type 1 assay by approximately 40% [78]. It has been suggested that these anti-*α*_2_ antibodies could be used as human angiogenesis inhibitors in tumour therapy [9].

### 6.1. Disintegrins and *α*_2_ *β*_1_

Small peptide agonists of integrins derived from protein fragments and snake venom are known to bind to *α*_2_ *β*_1_ in particular, thus presenting an exciting avenue for therapeutic intervention. These disintegrins usually contain the RGD motif, although this is not always the case [79]. Disintegrins such as angiocidin, a thrombospondin-1 binding protein, have been demonstrated to have antitumour effects by inhibiting angiogenesis. Importantly, angiocidin binds to *α*_2_ *β*_1_ and type I collagen with high affinity [80] and has been shown to bind to prostate, breast, melanoma, and colon cancer cell lines expressing *α*_2_ *β*_1_. Furthermore, expression levels of angiocidin in human colon cancer tissues have been shown to correlate with disease burden [80]. 

Similarly, snake venom derived disintegrins such as rhodocytin and jararhagin prevent angiogenesis. Both rhodocytin and jararhagin have a high affinity for *α*_2_ *β*_1_, and the former has been demonstrated to completely block the adhesion of fibrosarcoma cells to type 1 collagen preventing signalling [81], perhaps decreasing the amount of crosstalk between integrins. These molecules have been shown to have antitumour activity in *in vitro* and *in vivo* models; however, both are yet to be tested in prostate cancer models. Importantly, both rhodocytin and jararhagin are soluble, have small-molecular-weights, and by nature are able to move efficiently through tissue and the ECM [81]. Furthermore, E7820 is a small molecular weight inhibitor of integrin *α*_2_ *β*_1_ that has reached Phase II of human trial [82]. An aromatic sulphonamide derivative, E7820, has been shown to inhibit tubule formation in human umbilical vascular endothelial cells (HUVEC) by binding to, and inhibiting the expression of, the *α*_2_ subunit [83]. Phase II human trials are currently underway, which aim to examine the combinatory effect of E7820 with the traditional chemotherapeutic agent FOLFIRI (FOL-folinic acid, F-5-fluorouracil, and IRI-Irinotecan), and also E7820 with the monoclonal antibody cetuximab (Erbitux), in patients with metastatic colorectal cancer. In addition, the utilisation of *α*_2_ expression as a biomarker for E7820 efficacy is currently underway [84, 85]. The results of this study indicated that a relatively low knockdown of *α*_2_ expression achieved tumour stasis at biologically apposite levels for human administration [84]. 

## 7. Conclusion

The integrins *α*_2_ and *α*_6_ have emerged as putative biological markers for evaluation of prostate cancer susceptibility and also progression and metastasis. The combination of cell surface receptor coupled with differential expression is particularly important for the elucidation of the mechanisms that drive prostate cancer pathogenesis. The identification of the genetic drivers of tumour development, through use of new high-throughput genetic technologies combined with our knowledge of the biology of prostate cancer, is opening the door for the generation of new patient tailored therapies. However, whilst more than 40 disease susceptibility loci have been identified, the majority of loci identified confer only a modest risk and collectively account for approximately 30% of heritable disease burden; thus, further elucidation of the underlying genetic contributors is required. Inclusion of both common and rare prostate cancer variants in polygenic risk calculators is likely to improve their predictive value in diagnosis and prognosis.

Differential expression of the *α*_2_ integrins in prostate tumour cells has made them useful targets for both imaging techniques and therapeutics. Radiolabelled peptides, which ligate integrin *α*_2_ *β*_1_, have recently been developed for PET scanning with great success, allowing precise imaging of prostate tumour cells to occur in real time. The correlation with *α*_2_ *β*_1_ expression and prostate cancer has been a primary target in therapeutics. By targeting *α*_2_ *β*_1_, angiogenesis can be inhibited by preventing ligation with type 1 collagen, which is required for the formation of new blood vessels. Furthermore, the expression of *α*_2_ on platelets has been successfully utilised as a biomarker to examine the efficacy of an agonist targeting *α*_2_ in tumour cells in phase I and II human trials. Thus the existing knowledge of integrin biology and in particular the identification of *α*_2_ and *α*_6_, as key drivers of prostate cancer pathogenesis, is facilitating translation into new diagnostic and therapeutic applications. Further work is required to gain a thorough understanding of the genetic drivers identified for prostate cancer and how they influence tumour biology. This will permit informed application of our genetic discoveries in the clinical setting. 
